# Metal–Organic Framework Nanocarriers for Drug Delivery in Biomedical Applications

**DOI:** 10.1007/s40820-020-00423-3

**Published:** 2020-05-02

**Authors:** Yujia Sun, Liwei Zheng, Yu Yang, Xu Qian, Ting Fu, Xiaowei Li, Zunyi Yang, He Yan, Cheng Cui, Weihong Tan

**Affiliations:** 1 Molecular Science and Biomedicine Laboratory (MBL), State Key Laboratory of Chemo/Biosensing and Chemometrics, College of Chemistry and Chemical Engineering, College of Biology, Aptamer Engineering Center of Hunan Province, Hunan University, Changsha, 410082 People’s Republic of China; 2 Center for Research at Bio/Nano Interface, Department of Chemistry and Department of Physiology and Functional Genomics, UF Health Cancer Center, UF Genetics Institute and McKnight Brain Institute, University of Florida, Gainesville, FL 32611 USA; 3 Department of Electrical Engineering, Stanford University, Stanford, CA 94305 USA; 4 Institute of Molecular Medicine (IMM), Renji Hospital, State Key Laboratory of Oncogenes and Related Genes, Shanghai Jiao Tong University School of Medicine and College of Chemistry and Chemical Engineering, Shanghai Jiao Tong University, Shanghai, 200240 People’s Republic of China; 5 Institute of Cancer and Basic Medicine (IBMC), Chinese Academy of Sciences, The Cancer Hospital of the University of Chinese Academy of Sciences, Hangzhou, 310022 People’s Republic of China; 6 Foundation for Applied Molecular Evolution, 13709 Progress Boulevard, Alachua, FL 32615 USA

**Keywords:** Metal–organic frameworks, Drugs, Biomolecules, Drug delivery, Biomedical applications

## Abstract

Investigation of metal–organic frameworks (MOFs) for biomedical applications has attracted much attention in recent years. MOFs are regarded as a promising class of nanocarriers for drug delivery owing to well-defined structure, ultrahigh surface area and porosity, tunable pore size, and easy chemical functionalization. In this review, the unique properties of MOFs and their advantages as nanocarriers for drug delivery in biomedical applications were discussed in the first section. Then, state-of-the-art strategies to functionalize MOFs with therapeutic agents were summarized, including surface adsorption, pore encapsulation, covalent binding, and functional molecules as building blocks. In the third section, the most recent biological applications of MOFs for intracellular delivery of drugs, proteins, and nucleic acids, especially aptamers, were presented. Finally, challenges and prospects were comprehensively discussed to provide context for future development of MOFs as efficient drug delivery systems.

## Highlights

- Recent advances in biomedical applications of metal–organic framework (MOF) nanocarriers for drug delivery are summarized.
- State-of-the-art strategies to functionalize MOFs with therapeutic agents, as well as their merits and drawbacks, are comprehensively discussed.

## Graphical abstract

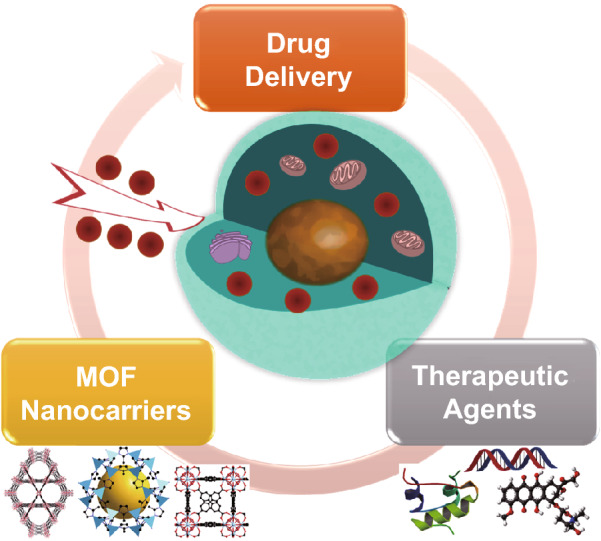

## Introduction

Metal–organic frameworks (MOFs) represent a promising class of highly ordered crystalline porous coordination polymers (PCPs) [1–3]. The extended infinite one-/two-/three-dimensional networks of MOFs are formed by the linkage of inorganic metal (e.g., transition metal and lanthanide metal) ions/clusters as the node and organic ligands (e.g., carboxylates, phosphonates, imidazolates, and phenolates) as the strut. In 1995, the Yaghi group studied selective binding and removal of guest molecules in a microporous MOF composed of 1,3,5-benzenetricarboxylate (BTC) and cobalt cation [4]. In 1999, the same group reported the design and synthesis of MOF-5, which contains 1,4-benzenedicarboxylate (BDC) and Zn_4_O clusters [5]. MOF-5 showed exceptionally high Langmuir surface area of 2900 m^2^ g^−1^. Over the past two decades, owing to extremely high surface area and pore volume, as well as tunable pore size and chemical composition, MOFs have been studied for various applications, including, for example, gas storage and separation [6–9], chemical separation [10, 11], catalysis [12–15], sensing [16–19], semiconductors [20], and bioimaging [21, 22].

In recent years, biomedical applications of MOFs for drug delivery have attracted increasing attention. When the size of MOF particles was scaled down to nanoscale, these nano-MOFs (NMOFs) can act as efficient nanocarriers to deliver agents for imaging, chemotherapy, photothermal therapy, or photodynamic therapy [23–27]. So far, various nanoparticle-based systems have been studied for drug delivery, such as liposomes, micelles, dendrimers, microbubbles, and solid particles [28]. The properties of MOF nanoparticles, dendrimers, and mesoporous silica nanoparticles are summarized in Table 1. Compared to other porous materials, MOFs show several outstanding advantages, such as (1) high surface area and porosity for high loading of therapeutic agents and (2) facile modification of physical (e.g., pore size and shape) and chemical properties of MOFs through inorganic clusters and/or organic ligands. For example, some MOFs containing lanthanide metals emit fluorescence under ultraviolet irradiation [17, 29]. In addition, desired functional groups can be added onto the organic ligands by predesigning of the ligands or post-synthetic modification approaches [30–32]. Other merits of MOFs include (3) diffusion of substrates to interact with the incorporated molecules via the MOF's open windows and pores; (4) moderate strength of coordination bonds, making MOFs biodegradable, and (5) well-defined structures beneficial for host–guest interaction studies [33]. With these unique properties, MOFs have been considered as one of the best candidates for drug delivery and cancer therapy.

**Table 1:** Summary of the properties of MOF nanoparticles, dendrimers, and mesoporous silica nanoparticles

	MOFs	Dendrimers	Mesoporous silica	Refs
Synthesis	Solvothermal, microwave, ultrasound	LBL process	Sol–gel process, hydrothermal	[163, 164] [165]
Morphology	Spherical, ellipsoidal, cubic, hexagonal, octahedral, etc.	Spherical	Spherical, cylindrical	[164–166]
Size distribution	Mono-/poly-disperse	Monodisperse	Mono-/poly-disperse	[167–169]
Pore shape	Highly tunable	Open internal cavity	Hexagonal, cubic	[166, 168, 170]
Pore feature	Amphiphilic	Hydrophobic	Hydrophobic	[33, 171, 172]
Structural tunability	Highly tunable through inorganic clusters and organic ligands	Depends on generation number and building blocks	Depends on synthetic conditions	[173–175]
Structural flexibility	Highly flexible	Depends on generation	Rigid	[164, 176, 177]

So far, a series of molecules have been selected as therapeutic agents to investigate MOFs for drug delivery applications. For instance, anticancer drugs including doxorubicin [34–37], cisplatin [38], topotecan [39], camptothecin [40], and 5-fluorouracil [41] have been incorporated into MOFs for intracellular delivery and cancer treatment. Some near-infrared region (NIR) organic dyes were used for photothermal therapy [42, 43]. MOFs functionalized with photosensitizers for photodynamic therapy (PDT) were also developed [44, 45]. In addition, delivery of many biomolecules by MOF nanocarriers have been studied in recent years [46]. Biomolecules exist in organisms and are critical to biological processes. They include macromolecules, e.g., nucleic acids, proteins, lipids, carbohydrates, and small molecules, e.g., amino acids and fatty acids. Delivery of these molecules with essential biological functions as biomolecular drugs provides a novel route for disease treatment.

In this review, we present the most recent progress of MOFs as promising nanocarriers for drug delivery in biomedical applications. First, we summarize state-of-the-art strategies to functionalize MOFs with therapeutic agents, including surface adsorption, pore encapsulation, covalent binding, and functional molecules as the building block. Then, we discuss recent biomedical applications of MOF nanocarriers for intracellular delivery of drugs, nucleic acids, and proteins. Finally, challenges and prospects are summarized in anticipation that this review can provide guidance for future researchers to engineer and explore MOFs as novel drug delivery systems for biological applications.

## Functionalization for Drug Delivery

MOFs exhibit unique properties, e.g., highly ordered structure, high surface area, and large pore volume, that enable them to adsorb functional molecules on their external surface or open channels, as well as trap these molecules inside the framework. In addition, functional molecules can be incorporated into MOFs through covalent bonds by one-pot synthesis or post-synthetic modification. In this part, we focus on four advanced strategies to functionalize MOFs with therapeutic agents for biological applications. They include surface adsorption, pore encapsulation, covalent binding, and functional molecules as the building block. Assessments of the merits and drawbacks of these approaches are also highlighted.

### Surface Adsorption

Due to high surface area and porosity, functional molecules can be adsorbed on the surface of MOFs. Generally, surface adsorption is achieved by stirring the pre-synthesized MOFs in a solution of functional molecules. Van der Waals interaction, *π*–*π* interaction, and hydrogen bonding are the dominant forces involved in this method. There is no strict requirement on the pore size or type of functional groups of MOFs for application of this relatively straightforward strategy. However, the leaching problem can hardly be avoided based on the weak interaction forces between molecules and MOF framework.

**Fig. 1:**
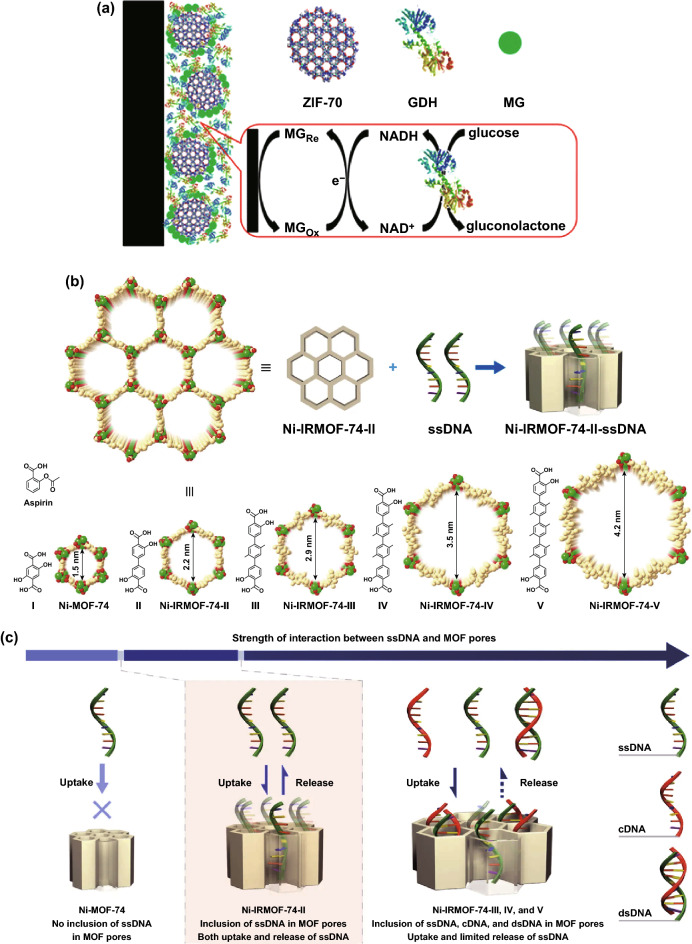
**a** Schematic illustration of fabricating an integrated electrochemical biosensor for glucose using ZIF-70 as the matrix to co-immobilize MG and GDH onto the surface of electrode. Reproduced with permission from Ref. [51]. Copyright 2013, American Chemical Society. **b** Schematic illustration of ssDNA immobilization in Ni-IRMOF-74 series with precisely controlled channel size. Ni, C, and O atoms were labeled with green, gold, and red color, respectively. **c** Gradual increase in the interaction between ssDNA and MOFs with the increase in MOF channel size. Relatively weak interactions ensured the uptake, protection, and reversible release of ssDNA. Reproduced with permission from Ref. [53]. Copyright 2018, Nature Publishing Group

Surface adsorption has been widely applied for enzyme immobilization [47]. In 2006, the Balkus group reported physical adsorption of microperoxidase-11(MP-11) catalyst on a nano-crystalline Cu-based MOF while maintaining the catalytic activity of MP-11 [48]. Compared to five mesoporous benzene silica (MBS) host materials, MP-11 supported on Cu-MOF showed better catalytic activity. Similarly, Liu et al. synthesized enzyme–MOF bioreactors for catalysis using MOFs with no chemical modification on the surface. Studies showed that host–guest interactions are mainly facilitated by hydrogen bonding and *π*–*π* interaction [49, 50]. Ma et al. investigated zeolitic imidazolate frameworks (ZIFs) as the matrix to co-immobilize methylene green (MG) and glucose dehydrogenase (GDH) to fabricate an integrated electrochemical biosensor (Fig. 1a) [51]. Among a series of ZIFs with different pore sizes, surface areas, and functional groups, ZIF-70 exhibited the best adsorption capacity for MG and GDH.

In addition to enzymes, nucleic acids can be immobilized on MOFs through surface adsorption [52]. For example, the Zhou and Deng group designed four isoreticular MOFs (Ni-IRMOF-74-II to -V) with tuned open channel size increased from 2.2 to 4.2 nm to precisely include single-stranded DNA (ssDNA, 11–53 nt) (Fig. 1b, c) [53]. The MOF framework acted as an excellent host to protect ssDNA from degradation by confining the nucleic acid chain completely inside the channel. Studies suggested that van der Waals interactions provided by suitable channel size and moderate accommodation in Ni-IRMOF-74-II are responsible for the reversible uptake and release of ssDNA. Subsequently, the Ni-IRMOF-74 series were applied as nonviral vectors for intracellular delivery and gene silencing. Two MOFs (Ni-IRMOF-74-II and -III) with weaker interactions exhibited optimal transfection efficiency in mammalian immune cells, 92% in primary mouse immune cells (CD4^+^ T cell) and 30% in human immune cells (THP-1 cell), over commercial agents (Lipo and Neofect).

### Pore Encapsulation

Since MOFs possess high porosity and pores tunable from microporous to mesoporous, many types of functional molecules can be accommodated inside the pores. As a host material, MOFs prevent the loaded substrates from leaching, as well as providing them a protective environment against external adverse factors.

A versatile and efficient way to incorporate functional molecules into MOFs is pore encapsulation through de novo synthesis. During the synthetic process, MOF formation and substrate encapsulation occur at the same time. As a result, this method enables immobilization of larger molecules, compared to the pore size of MOFs, into the cavity of MOFs. However, it requires that the substrate is stable under synthetic conditions.

So far, this method has been widely used to encapsulate anticancer drugs inside the MOF host for intracellular delivery and subsequent release [54]. For instance, monodisperse ZIF-8 nanospheres of uniform particle size (70 nm) were synthesized with the anticancer drug camptothecin encapsulated inside the framework [40]. Enhanced cell internalization and reduced cytotoxicity were demonstrated by studies on the MCF-7 breast cancer cell line. By mixing inorganic metal salts, organic ligands, and drug molecules, the anticancer drug 3-methyladenine was successfully incorporated into ZIF-8 [55]. An increased efficiency of autophagy inhibition was observed in HeLa cells treated with 3-methyladenine@ZIF-8 nanoparticles. ZIF-8 has been considered as an ideal host material for intracellular drug delivery owing to good monodispersity, optimal size for cellular uptake, ease of synthesis under mild environment, and ease of surface modification.

**Fig. 2:**
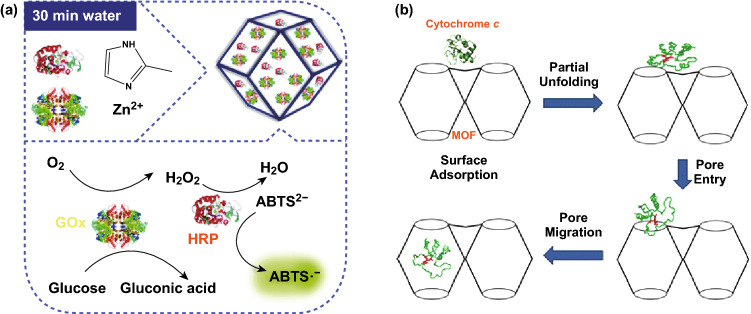
**a** One-step synthesis of ZIF-8 nanocrystals embedding multiple enzymes GOx and HRP. Reproduced with permission from Ref. [56]. Copyright 2015, The Royal Society of Chemistry. **b** Mechanism of Cyt c translocation into the MOF interior through relatively small windows. Reproduced with permission from Ref. [59]. Copyright 2012, American Chemical Society

In addition to anticancer drugs, enzyme encapsulation by de novo synthesis has been accomplished. Wu et al. reported a one-step and facile synthesis of ZIF-8 nanocrystals containing glucose oxidase (GOx) and horseradish peroxidase (HRP) in aqueous solution at 25 °C (Fig. 2a) [56]. The prepared GOx&HRP@ZIF-8 bioconjugates exhibited high stability, selectivity, and catalytic efficiency. Hou et al. encapsulated GOx into magnetic ZIF-8 to construct a reusable mimic multi-enzyme system [57].

In general, synthetic conditions, e.g., high temperature, organic solvents, and acidic environment, of MOFs are too harsh for biomolecules, such as enzymes, to maintain their structural features and activities. To address this issue, pore encapsulation by a post-synthetic modification strategy provided a powerful route to incorporate biomolecules under mild conditions. In 2011, the Ma group reported immobilization of microperoxidase-11 (MP-11) into a mesoporous MOF, denoted as Tb-mesoMOF [58]. By immersing freshly synthesized Tb-mesoMOF crystals in MP-11 solution, the enzyme with dimensions of about 3.3 × 1.7 × 1.1 nm^3^ was successfully loaded into MOFs containing cages of 3.9 and 4.7 nm in diameter. MP-11@Tb-mesoMOF exhibited higher catalytic activity compared to mesoporous silica material (MCM-41). Later, the group found that cytochrome c (Cyt c) with dimensions of 2.6 × 3.2 × 3.3 nm^3^ could be trapped by a MOF with smaller window sizes (1.3 and 1.7 nm) [59]. Mechanistic studies suggested that the enzyme was flexible and could change its conformation significantly to pass through small nanopores to enter the MOF's interior (Fig. 2b). Similarly, the Zhou group prepared stable PCN-333 containing large mesoporous cages as single-molecule traps (SMTs) for enzyme encapsulation, which prevented enzymes from aggregation and leaching [60]. Three different enzymes were successfully encapsulated into PCN-333(Al) with record-high loading and recyclability.

### Covalent Binding

Although various functional molecules have been incorporated into MOFs by surface adsorption and pore encapsulation methods, relatively weak interaction forces between these molecules and MOFs often result in slow leaching problems. Considering this, immobilization through covalent binding provides a feasible solution.

**Fig. 3:**
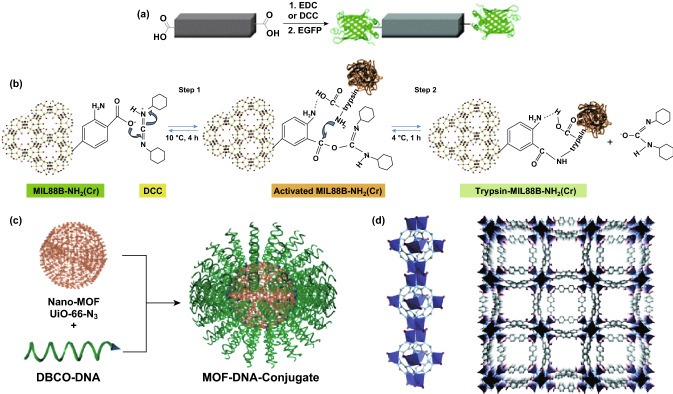
**a** Activation of the 1D-polymer, [(Et_2_NH_2_)(In(pda)_2_)]_n_, with EDC or DCC to conjugate with EGFP. Reproduced with permission from Ref. [62]. Copyright 2011, The Royal Society of Chemistry. **b** DCC activation of MIL-88B-NH_2_(Cr) and following trypsin immobilization. Reproduced with permission from Ref. [63]. Copyright 2012, Wiley-VCH. **c** Schematic illustration of surface functionalization of UiO-66-N_3_ nanoparticles with DNA through a click reaction with DBCO-DNA. Reproduced with permission from Ref. [68]. Copyright 2014, American Chemical Society. **d** The crystal structure of bio-MOF-1. The MOF consists of zinc—adeninate columns linked together into a 3D framework by BPDC linkers to generate a material with 1D channels. Reproduced with permission from Ref. [77]. Copyright 2009, American Chemical Society

In general, the MOF surface possesses many kinds of functional groups, such as amino, carboxyl, and hydroxyl group, that can be utilized to form covalent bonds with the reactive groups on the target [61]. For instance, Jung et al. reported conjugation of enhanced green fluorescent protein (eGFP) and Candida antarctica lipase B (CAL-B) enzyme on the MOF surface through post-synthetic modifications [62]. A coupling reagent, such as 1-ethyl-3-(3-dimethylaminopropyl) carbodiimide (EDC) or dicyclohexyl carbodiimide (DCC), was used to activate the dangling carboxylate groups of organic ligands on the MOF surface for subsequent bioconjugation (Fig. 3a). Studies showed that enantioselectivity and activity in transesterification of ( ±)-1-phenylethanol were well preserved for CAL-B-MOF bioconjugates. With a similar coupling method, the protease enzyme trypsin was successfully immobilized onto MIL-88B(Cr), MIL-88B-NH_2_(Cr), and MIL-101(Cr) [63]. This was achieved by nucleophilic attack of the amine groups of trypsin on DCC-activated MOFs (Fig. 3b). Trypsin-MIL-88B-NH_2_(Cr) exhibited bovine serum albumin (BSA) protein digestion efficiency comparable to that of native trypsin digestion. In addition to the carboxylate group, the amino group on organic ligands can be used to coordinate with enzymes, such as glucose oxidase [64] and soybean epoxide hydrolase [65].

Click reaction on the organic linkers has been utilized for immobilization of biomolecules [66, 67]. The Mirkin group reported the first examples of nucleic acid–MOF nanoparticle conjugates [68]. They were synthesized by a strain promoted click reaction between azide-functionalized UiO-66 and dibenzylcyclooctyne-functionalized DNA (Fig. 3c). Since the pore size of UiO-66 is small, the DNA strands were coordinated to the external surface of MOF nanoparticles. The UiO-66 structure could be maintained during the chemical reaction. Compared to nonfunctionalized MOF nanoparticles, the DNA–MOF conjugates exhibited higher colloidal stability and enhanced cellular uptake.

In addition to organic linkers, inorganic metal clusters provide another type of reactive sites in MOFs to covalently bind functional molecules. In 2017, the Mirkin group reported a general and direct approach to functionalize the external surface of MOF nanoparticles with oligonucleotides [69]. Through this coordination chemistry-based strategy, the external metal nodes of MOF nanoparticles were covalently linked with terminal phosphate-modified oligonucleotides. Nine different archetypical MOFs containing different metals (Zr, Cr, Fe, and Al) were all successfully modified with oligonucleotides on the external surface. This method allows functionalization of the particle surface independent of MOF structure. In addition, DNA is chemically programmable to manipulate interparticle interactions. The prepared nucleic acid-nanoparticle conjugates could be used for DNA mediated programmable assembly and intracellular process manipulation. The Zhou group reported a facile one-pot approach to incorporate a series of porphyrin derivatives into stable Zr-MOFs, taking advantage of the available coordination sites on Zr_6_ clusters [70]. By mixing ligands of different geometries and connectivities, tunable amounts of tetratopic tetrakis(4-carboxyphenyl)porphyrin (TCPP) ligands were successfully integrated, while, at the same time, maintaining the crystal structure, morphology, and ultrahigh chemical stability of the parent MOF. This strategy provided a facile route toward multifunctional stable Zr-MOFs for potential applications.

### Functional Molecules as the Building Block

Another approach to functionalize MOFs is designing functional molecules as the building block. Biomolecules generally contain several reactive chemical groups that can coordinate with inorganic metals. So far, amino acids [71], peptides [72, 73], nucleobases [74], and saccharides [75] have been applied as the organic ligands to synthesize bio-MOFs. Bio-MOFs tend to have better biocompatibility and special biological functionality. However, most biomolecules are highly flexible with low symmetry, making it a challenge to use them directly to form high-quality MOF crystals.

For nucleobases, several oxygen and nitrogen atoms in the structure are accessible as lone pair electron donors to coordinate with metal ions. Among nucleobases, adenine has been widely studied to build bio-MOFs owing to rich binding modes provided by four N atoms in the purine ring and one exocyclic amino group [76]. In order to form a highly ordered MOF structure with low-symmetry adenine as the building block, a symmetrical co-ligand was introduced to guide the synthesis. By mixing biphenyldicarboxylic acid (BPDC), adenine, and zinc acetate dihydrate, the Rosi group synthesized crystalline and porous bio-MOF-1 with the following formula: Zn_8_(ad)_4_(BPDC)_6_O·2Me_2_NH_2_·8DMF·11H_2_O [77]. The MOF consists of infinite 1D zinc-adeninate columns comprising corner-fused zinc-adeninate octahedral building units (ZABUs), which are interconnected by linear BPDC linkers (Fig. 3d). Later, the same group reported a mesoporous MOF named as bio-MOF-100 (Zn_8_(ad)_4_(BPDC)_6_O_2_·4Me_2_NH_2_·49DMF·31H_2_O) with higher surface area (4300 m^2^ g^−1^) and pore volume (4.3 cm^3^ g^−1^) [78]. The MOF consists of discrete ZABUs interconnected with BPDC linkers. Each ZABU is connected to four neighboring ZABUs via 12 BPDC linkers to generate a three-dimensional structure with large cavities and channels. The Zhou group added a highly symmetrical co-ligand to obtain Zn_3_[Zn_2_(μ_2_-H_2_O)]_3_(Ad)_6_(TATB)_4_(DMF), (Ad = adeninate, TATB = 4,4′,4′'-s-triazine-2,4,6-triyl-tribenzoate) (PCN-530) [79].

## Applications in Drug Delivery

One of the major problems for conventional chemotherapy is the need to use a high drug dose as a consequence of poor biodistribution, resulting in frequent dose-related side effects [80]. This calls for the exploration of novel and efficient drug delivery systems (DDSs). Recent studies have shown the application of MOF nanocarriers to achieve targeted drug delivery, increased cellular uptake, and controlled drug release, making MOFs a promising class of DDSs for drug delivery, including anticancer drugs, antimicrobial agents, metabolic labeling molecules, antiglaucoma medication, and hormone [42, 54, 81–84]. So far, targeted delivery has been achieved by three strategies, including passive delivery owing to enhanced permeability and retention (EPR) phenomenon, active/ligand delivery (e.g., folic acid, antibody, and hyaluronic acid), and triggered delivery (e.g., pH, photoirradiation, temperature, and pressure). Pinocytosis includes clathrin-mediated endocytosis, caveolin-mediated endocytosis, as well as clathrin- and caveolin-independent endocytosis [85, 86]. During the clathrin-mediated endocytosis, receptors are responsible for cargo recognition, followed by the formation of clathrin-coated vesicles, which are usually up to 200 nm in size [87]. These vesicles merge with early endosomes, then mature into late endosomes. The late endosomes fuse with lysosomes, which leads to the hydrolysis of the DDS and the cargo, consequently diminishing its therapeutic effect [88]. On the other hand, caveolin-mediated endocytosis involves the formation of lipid raft-enriched flask-shaped invaginations coated with caveolin [89, 90]. Nanoparticles internalized through caveolin-mediated endocytosis can be delivered later to different locations inside the cell. Premature drug release is a major drawback for many MOF-based DDS. Recent studies have shown that several strategies such as encapsulation of drugs into MOFs by a one-pot synthesis, surface coating of MOFs, and triggered drug release could be applied to overcome this problem. In this section, we mainly focus on recent progress in biomedical applications of MOFs for the delivery of drugs, nucleic acids, and proteins.

### Drugs

Because of their extremely large surface area, highly porous structure, and easy chemical modification, MOFs have been extensively studied as ideal nanocarriers to load various drugs. For example, doxorubicin hydrochloride (DOX) represents one of the first-line chemotherapeutic drugs for breast cancer, ovarian cancer, and lymphoblastic leukemia [91]. Busulfan (Bu) is an amphiphilic antitumor drug widely used in chemotherapy for leukemia as an alternative to total-body irradiation [92]. Topotecan (TPT) is a derivative of the drug camptothecin (CPT) that is clinically used for treatment of refractory ovarian [93] and small cell lung cancers [94]. In general, drugs are loaded into MOFs by in situ encapsulation or a post-synthetic modification strategy. The former is a relatively straightforward method suitable for thermostable drugs to overcome premature drug release. Although more complicated and time-consuming, the latter provides a milder environment to avoid destroying drug molecules. With the development of MOF chemistry, a series of MOFs have been explored as promising candidates for application in this area [95]. We selected some MOFs as examples (e.g., ZIF-8, MIL-100 and MIL-101) in this part to summarize recent progress of MOFs as a novel class of nanocarrier for drug delivery. The information of these MOFs (e.g., surface area and pore volume), the agents delivered, and the cells/animals used to test the DDS were summarized in the table (Table 2). Strategies to enhance therapeutic efficiency by, for example, increasing the drug loading, facilitating the cellular uptake and controlling the drug release are also discussed.

**Table 2:** Summary of the MOF type, surface area, pore size, agents delivered, and cells/animals tested for drug delivery discussed in this review

MOF	BET surface area (m^2^ g^−1^)	Pore size (Å)	Agents delivered	Cells/animals tested	Refs
ZIF-8	1300	12	DOX	MCF-7 cells	[34]
			DOX	MDA-MB-468 cells	[103]
			DOX	B16F10 bearing mice	[106]
			Camptothecin	MCF-7 cells	[40]
			3-MA	HeLa cells	[55]
			Ceftazidime	Escherichia coli	[83]
			pEGFP-C1	MCF-7 cells	[127]
			VEGF aptamer/insulin/GOx	MCF-10A cells	[149]
			DOX/BSA	MCF-7 cells	[152]
MIL-100	1800	25 and 29	ICG	MCF-7 cells	[42]
			_D_-AzAla	MRSA-bearing mice	[81]
			Brimonidine tartrate	661 W cells	[84]
			DOX	HepG-2 cells	[36]
			Topotecan	PANC1 cells	[39]
MIL-101	2200	29 and 34	Cisplatin	HT-29 cells	[112]
			DOX	H22 tumor-bearing mice	[113]
			siRNAs	MCF-7/T cells	[130]
NU-1000	2200	12 and 30	Insulin	N.A	[82]
			Insulin/DNA	SK-OV cells	[151]
UiO-66	1200	8 and 12	Tamra-labeled DNA	HeLa cells	[68]
			AS1411/DOX	MCF-7 cells	[147]
UiO-67	2300	12 and 16	Brimonidine tartrate	661 W cells	[84]
UiO-68	4000	16 and 20	Cisplatin/siRNAs	SKOV-3 cells	[129]
			ATP/AS1411	MDA-MB-231	[148]
PCN-333	4000	42 and 55	Tyrosinase	RPMI-1640	[158]
ZIF-90	1300	11	Cas9	HeLa cells	[153]

Zeolite imidazolate frameworks (ZIFs) are a subclass of MOFs that have been applied in gas separation [96], chemical separation [97], and as carriers for metal nanoparticles [98] and drugs [99]. ZIF-8 contains zinc ions and 2-methylimidazolate. Based on its high thermal and hydrothermal stability, as well as nontoxic and biocompatible characteristics, ZIF-8 has been regarded as a promising nanocarrier for drug delivery [100]. It is worth noting that ZIF-8 is stable under physiological conditions but unstable under acidic environments, making it feasible to use in a pH-responsive drug delivery system.

**Fig. 4:**
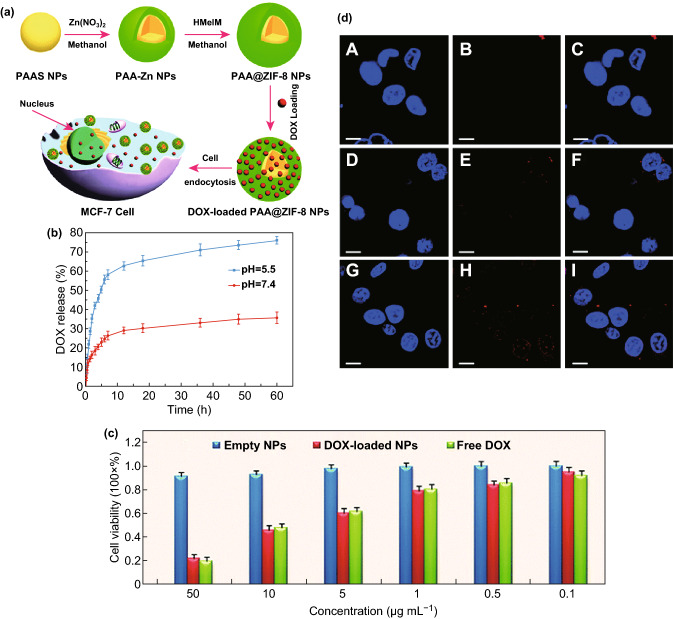
**a** Schematic illustration of the synthesis of PAA@ZIF-8 as the nanocarrier for DOX drug loading and pH-controlled release. **b** Drug release of DOX-loaded PAA@ZIF-8 at pH 5.5 and 7.4 at 37 °C. **c** In vitro cytotoxicity of PAA@ZIF-8, DOX-loaded PAA@ZIF-8, and free DOX against MCF-7 cells at different concentrations after 24 h. **d** CLSM images of MCF-7 cells incubated with DOX-loaded PAA@ZIF-8 ([DOX] = 20 μg mL^−1^) for 3 h (A–C), 12 h (D–F) and 24 h (G–I) at 37 °C, respectively. Columns 1–3 can be classified to cell nucleus (dyed in blue by Hoechst 33,342), DOX-loaded PAA@ZIF-8, and the merged images of both, respectively. All scale bars are 10 μm. Reproduced with permission from Ref. [34]. Copyright 2014, The Royal Society of Chemistry

In 2012, the Junior group successfully loaded ZIF-8 with DOX (4.9 wt%) by post-synthetically stirring dehydrated ZIF-8 powder with the drug in aqueous solution [101]. Highly controlled and gradual drug release was observed (66% drug release after 30 days). Similarly, ZIF-8 was used for 5-fluorouracil (5-FU) delivery as a pH-responsive drug delivery vehicle [102]. A remarkable capacity of the drug was achieved through post-synthetic modification of ZIF-8 with 5-FU, with around 660 mg of 5-FU/g of ZIF-8. Experiments suggested a faster drug release in a mild acidic buffer solution (pH = 5.0) compared to that in neutral condition (pH = 7.4). Later, the Su group reported fabrication of ZIF-8 with polyacrylic acid (PAA) to reach an ultrahigh DOX loading capability (1.9 g DOX/g MOF) using a facile and simple route (Fig. 4a) [34]. As a pH-dependent drug delivery vehicle, PAA@ZIF-8 released drugs faster under acidic conditions (pH = 5.5) (Fig. 4b). The standard 3-(4,5-dimethylthiazol-2-yl)-2,5-diphenyltetrazolium bromide (MTT) cell assay was performed on MCF-7 cells to characterize the cytotoxicity. Results showed that the cytotoxic efficacy of the DOX-loaded PAA@ZIF-8 nanoparticles was similar to that of the free DOX and enhanced by increased DOX concentration (Fig. 4c). The confocal laser scanning microscopy (CLSM) analyses suggested that increased amounts of DOX were delivered to the nucleus from 3 to 24 h (Fig. 4d).

**Fig. 5:**
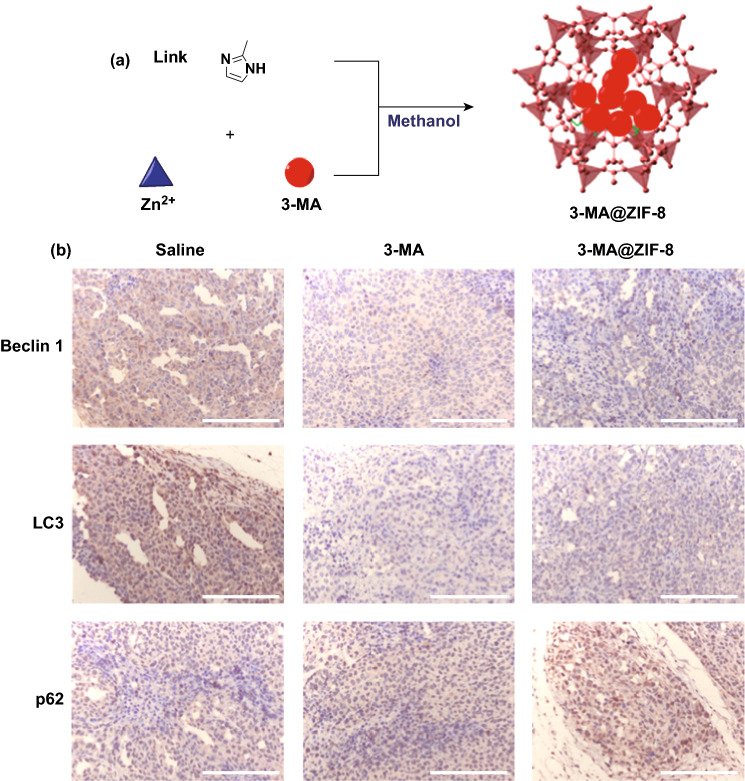
**a** Encapsulation of autophagy inhibitor 3-MA into ZIF-8 nanoparticles. **b** Autophagic regulation proteins of xenograft tumor estimated by immunohistochemistry (scale bar: 200 μm). Reproduced with permission from Ref. [55]. Copyright 2017, American Chemical Society

Compared to the post-synthetic modification method, the in-situ encapsulation approach avoids impeding the access of large molecules by small pore opening of MOFs and alleviates the problem of premature drug release. Tsung et al. developed a general synthetic strategy toward in situ incorporation of drug molecules (e.g., camptothecin) into the framework of ZIF-8 nanospheres for drug delivery [40]. Through their method, zinc nitrate, 2-methyl imidazole and drug molecules were mixed to generate uniform ZIF-8 nanoparticles (70 nm) with single-crystalline structure. The size of nanoparticles was optimized to facilitate cellular uptake. Enhanced MCF-7 cell death by camptothecin-encapsulated ZIF-8 nanoparticles was observed, indicating internalization and intracellular release of the drug. Using a similar strategy, an autophagy inhibitor, 3-methyladenine (3-MA), was encapsulated into ZIF-8 nanoparticles with a high loading (19.8 wt%) (Fig. 5a) [55]. TEM studies suggested that the cellular uptake of 3-MA@ZIF-8 into HeLa cells is facilitated through the nanoparticle internalization. The ZIF-8 nanoparticles were localized mainly in the cytoplasm and subcellular organelles. And the cells treated with 3-MA@ZIF-8 showed more autophagosomes than that of 3-MA. The xenograft tumor of cervical cancer HeLa cell was established to evaluate the antitumor effect of the nanoparticles. Next, the autophagic regulation proteins were estimated by immunohistochemistry. Compared with free 3-MA, 3-MA@ZIF-8 showed upregulating of p62 and downregulating of the autophagy-related markers, Beclin 1 and LC3 (Fig. 5b).

**Fig. 6:**
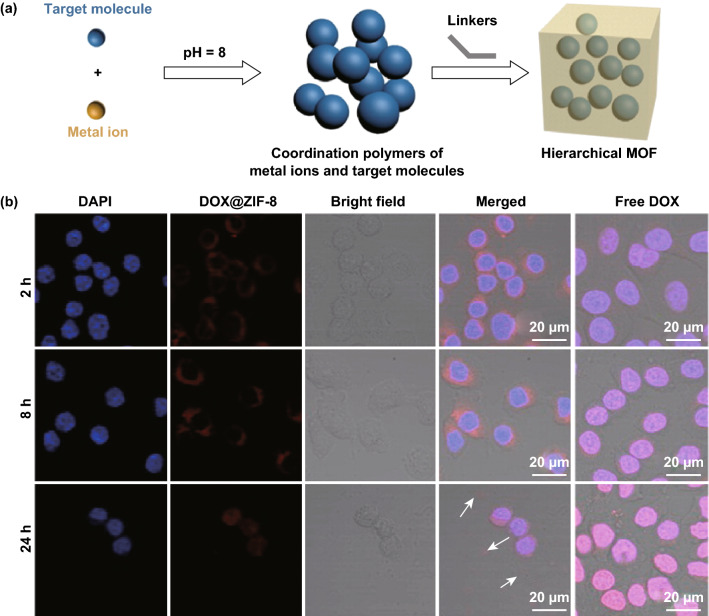
**a** pH-induced one-pot synthesis of hierarchical ZIF-8 with encapsulated drug molecules. **b** Cell uptake studies conducted to compare the localizations of DOX@ZIF-8 and free DOX in the MDA-MB-468 cells. Reproduced with permission from Ref. [103]. Copyright 2015, American Chemical Society

Unlike adding all the reactants at the same time, Zou and coworkers reported a novel pH-induced one-pot synthesis of DOX@ZIF-8 [103]. First, inorganic metal ions and drugs were self-assembled to form coordination polymers at pH = 8. Then, the organic linkers were added to disassemble metal ions from the drugs. As a result, drug molecules were encapsulated during MOF formation, generating hierarchical ZIF-8 (Fig. 6a). Confocal microscopy was used to compare the uptake of free DOX and DOX@ZIF-8 into the MDA-MB-468 cells (Fig. 6b). Results showed that free DOX entered the nuclei very fast (within 2 h) and accumulated in the nuclei. While DOX@ZIF-8 nanoparticles were initially observed in the cytoplasm. After 24 h, most of the cells treated with DOX@ZIF-8 were dead, and only cellular debris was observed. Compared with free DOX, DOX@ZIF-8 showed pH-responsive drug release and increased efficacy on breast cancer cell lines.

**Fig. 7:**
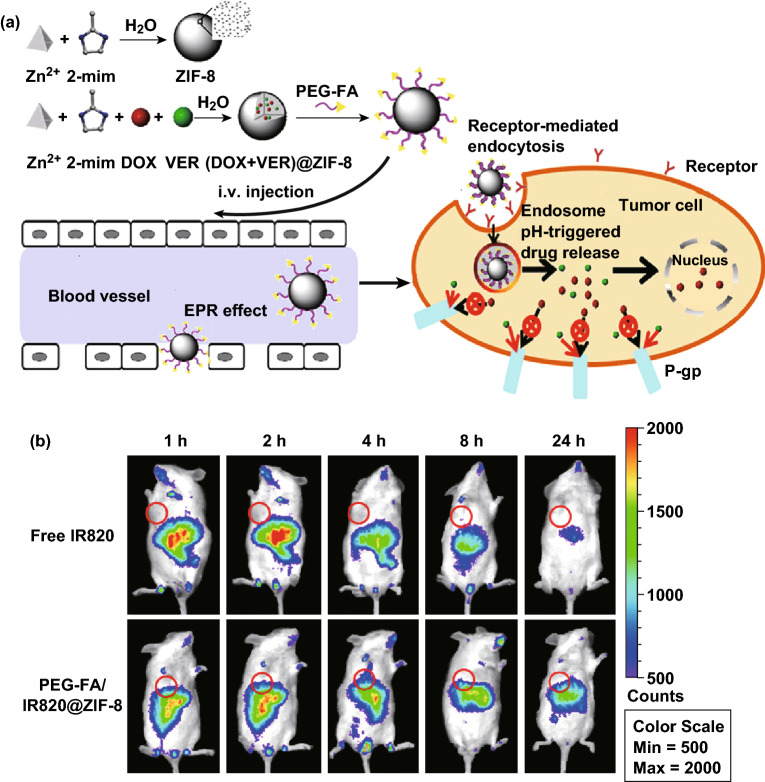
**a** Schematic representation of pH-responsive ZIF-8 as a co-delivery system for overcoming MDR for efficient targeted cancer therapy: PEG-FA/(DOX + VER)@ZIF-8 synthesis; accumulation in tumors via EPR effect; internalization via FR-mediated endocytosis; pH-dependent drug release under weak acidic environments; VER-mediated MDR reversal. The biological ligand has been binded (physically touch the receptor) in order for receptor-mediated endocytosis to take place. **b** In vivo fluorescence imaging of B16F10 bearing mice at 1, 2, 4, 8, and 24 h after the injection of free IR820 or PEG-FA/IR820@ZIF-8. Reproduced with permission from Ref. [106]. Copyright 2017, American Chemical Society

Multidrug resistance (MDR) has been reported as one major cause for the failure of cancer chemotherapy. The main reason for MDR is overexpression of active efflux transporters, e.g., *P*-glycoprotein [104, 105]. To address this issue, the Luan group reported the use of ZIF-8 as a co-delivery system for efficient targeted cancer therapy [106]. Generally, MOF nanoparticles accumulate preferentially in the neoplastic tissues through passive targeting owing to the EPR effect [107, 108]. The EPR effect is based on the size range of the MOF nanoparticles (12.5 to 150 nm) and two fundamental characteristics of the neoplastic tissues (the leaky vasculature and impaired lymphatic drainage). In this study, verapamil hydrochloride (VER) was selected as the *P*-glycoprotein inhibitor to overcome MDR, while DOX was selected as an anticancer drug. Through a facile one-pot process, VER and DOX were encapsulated into ZIF-8 to form uniform nanoparticles with high stability. Furthermore, ZIF-8 was stabilized by methoxy poly(ethylene glycol)-folate (PEG-FA) to realize prolonged circulation and active targeted drug delivery (Fig. 7a). According to the cell uptake and near-infrared fluorescence (NIRF) imaging results, drug accumulation in tumors was increased by PEG-FA/(DOX + VER)@ZIF-8. Studies revealed that both FR-mediated endocytosis and VER-mediated multidrug resistance reversal improved the internalization of DOX and enhanced its cytotoxicity for efficient anticancer effect. To study the targeted behaviors of PEG-FA/ZIF-8 in vivo, the mice bearing tumors derived from B16F10 cells were selected and monitored by NIRF optical imaging system (Fig. 7b). The mice injected with PEG-FA/IR820@ZIF-8 exhibited higher intensity of fluorescence at the tumor sites than that injected with free IR820. In addition to folic acid, other molecules have been studied for active delivery. For example, Cai et al. modified MOF nanoparticles with hyaluronic acid (HA) and indocyanine green (ICG) for imaging-guided, anticancer photothermal therapy (PTT) [42]. The in vitro and in vivo imaging showed that the MOF@HA@ICG exhibited greater cellular uptake in CD44-positive MCF-7 cells and enhanced tumor accumulation in xenograft tumors. Qi et al. developed a MOF-based platform modified with antiepithelial cell adhesion molecule (anti-EpCAM) antibody to achieve cell recognition and targeted capture [109]. The platform acted as an efficient trapper for targeted tumor cells (MCF-7 cells), exhibiting excellent capture capability and selectivity.

**Fig. 8:**
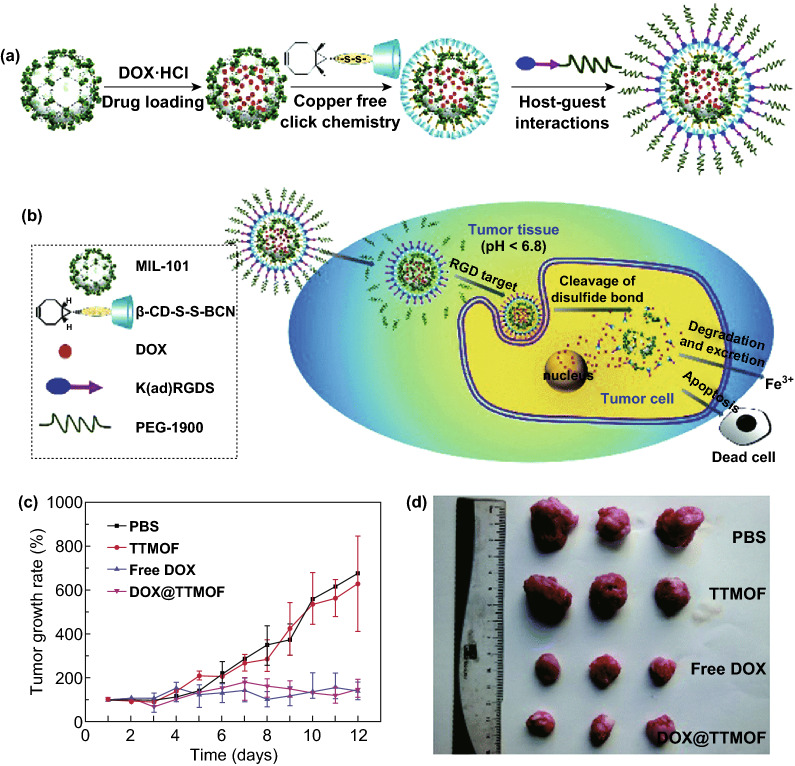
**a** Drug loading and post-synthetic surface modification of MIL-101. **b** Multifunctional MIL-101 as a dual-responsive DDS for tumor-targeted drug delivery and cancer therapy. **c** Tumor volume change in H-22 tumor-bearing mice after treatment. **d** Images of the tumor after 12 days. Reproduced with permission from Ref [113]. Copyright 2015, The Royal Society of Chemistry

MIL-101 is a zeotypic MOF built from trimers of metal octahedra and 1,4-benzenedicarboxylic acid (BDC) [110]. As an example of nontoxic porous iron(III)-based MOFs, MIL-101(Fe) has been selected for anticancer drug delivery studies owing to its biocompatible, biodegradable, and highly water-stable characteristics. Moreover, this mesoporous MOF possesses pores of 29 Å and 34 Å, holding great promise for high loading and sustained release of drugs [111]. For instance, ethoxysuccinato-cisplatin anticancer prodrug was post-synthetically loaded into MIL-101-NH_2_(Fe) [112]. Controlled cargo release was realized by surface coating with silica shell. Recently, Zhang et al. reported a one-pot and organic solvent-free "green" post-synthetic modification method to construct a dual-responsive, tumor targeting drug delivery system based on MIL-101(Fe) [113]. After incorporation of DOX, the surface of MOF was modified with a bicyclononyne (BCN)-functionalized β-cyclodextrin (β-CD) derivative (β-CD-SS-BCN) by copper-free click chemistry. Then, further modification with an α_v_β_3_ integrin-targeting peptide-functionalized polymer Lys(adamantane)-Arg-Gly-Asp-Ser-bi-PEG1900 (bi = benzoic imine bond, K(ad)RGDS-PEG1900) was carried out through host–guest interaction between β-CD and adamantane group (Fig. 8a). The obtained tumor targeting MOF-based DDS was abbreviated as TTMOF. In the in vivo experiments, hepatoma H22 tumor-bearing mice were treated with PBS, 5.0 mg kg^−1^ free DOX, 5.0 mg kg^−1^ DOX-loaded TTMOF, and 50 mg kg^−1^ empty TTMOF, respectively. Both DOX-loaded TTMOF and free DOX exhibited significant tumor growth inhibition (Fig. 8c, d). Studies demonstrated that premature drug release was efficiently prevented by multifunctional surface coating. In addition, enhanced tumor uptake and controlled drug release were achieved as a consequence of pH-responsive benzoic imine bond and redox-responsive disulfide bond. Later, exosome-coated MOF nanoparticles as a smart and efficient drug delivery system has been reported, which exhibited high therapeutic efficiency and no premature leakage [114].

In addition to anticancer drugs, delivery of other agents by MOF-based DDS have been achieved in recent years. For example, Mao et al. reported in vivo metabolic labeling of bacteria using MIL-100 (Fe) nanoparticles as the nanocarrier for precise delivery of 3-azido-_D_-alanine (_D_-AzAla) [81]. After intravenous injection of _D_-AzAla@MIL-100(Fe) into MRSA-bearing mice, followed by intravenous injection of DBCO-Cy5, the infected tissue of mice showed a significant DBCO-Cy5 accumulation. Later, Gallis et al. studied ZIF-8 as a robust platform to support the sustained release of ceftazidime, an important antimicrobial agent for many critical bacterial infections [83]. The antibacterial properties of ceftazidime@ZIF-8 were confirmed against Escherichia coli. This is the first study to unequivocally demonstrate direct internalization of MOFs using confocal microscopy via 3D reconstructions of z-stacks. Recently, investigation of MOF nanocarriers for intraocular incorporation of brimonidine tartrate to treat chronic glaucoma has been reported [84]. The cytotoxicity tests suggested low toxicity of the nanoparticles in retinal photoreceptor cells (661 W). The Farha group immobilized insulin in NU-1000 with a high loading (40 wt%) in 30 min [82]. Studies showed that the MOF capsules effectively prevented insulin from degrading in the presence of stomach acid and the digestive enzyme, pepsin.

**Fig. 9:**
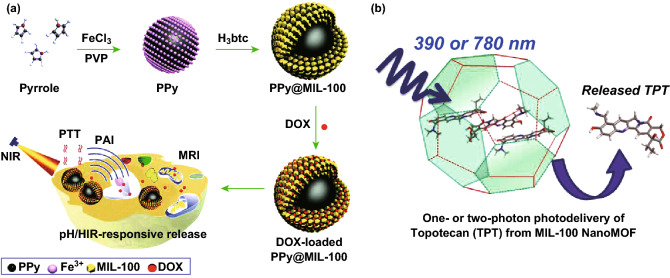
**a** Schematic illustration of the synthesis of PPy@MIL-100(Fe) as a pH/NIR-responsive drug carrier for dual-mode imaging and synergistic chemo-photothermal therapy. Reproduced with permission from Ref. [36]. Copyright 2017, The Royal Society of Chemistry. **b** MIL-100(Fe) NMOF for one- or two-photon-induced photodelivery of topotecan. Reproduced with permission from Ref. [39]. Copyright 2013, American Chemical Society

So far, several strategies have been investigated to enhance the therapeutic efficiency of MOF-based drug delivery systems [115]. MOF nanocarriers for increased drug loading, targeted drug delivery, facilitated cellular uptake, pH-responsive drug release, and multidrug resistance reversal were discussed in the previous examples. In recent years, triggered delivery has been widely studied as a powerful strategy to overcome premature drug release. For example, Wang et al. synthesized well-dispersed polypyrrole (PPy)@MIL-100(Fe) nanoparticles with a core–shell structure [36]. Upon DOX loading, this drug delivery system was employed for synergistic chemo-photothermal therapy for cancer cells based-on pH/NIR-responsive drug release (Fig. 9a). Similarly, light irradiation was utilized to induce drug release from MOFs. Douhal and coworkers encapsulated a hydrophilic anticancer drug (topotecan) inside MIL-100 NMOF in a "ship in a bottle" fashion [39]. They demonstrated that one- and two-photon light irradiation could promote stimuli-dependent drug release from the NMOFs (Fig. 9b). Remarkably, the formation of topotecan aggregates not only avoided burst release but also strongly stabilized MIL-100(Fe) against degradation. In addition to pH and light irradiation, other triggers have been explored for controlled drug release of MOFs, such as magnetic-responsive MOFs [116, 117], iron-responsive MOFs [118, 119], temperature-responsive MOFs [120, 121], pressure-responsive MOFs [122], humidity-responsive MOFs [123], and redox-responsive MOFs [124–126].

### Nucleic Acids

Nucleic acids represent a class of biomolecules that contains deoxyribonucleic acid (DNA) and ribonucleic acid (RNA), depending on the type of sugar moiety. Nucleic acids play an important role in the storage and expression of genetic information. In general, incorporation of nucleic acids into MOF nanocarriers could protect them against degradation and accelerate their cellular uptake. Moreover, surface modification of MOF nanoparticles with nucleic acids could increase their colloidal stability by providing steric and electrostatic hindrance to aggregation.

**Fig. 10:**
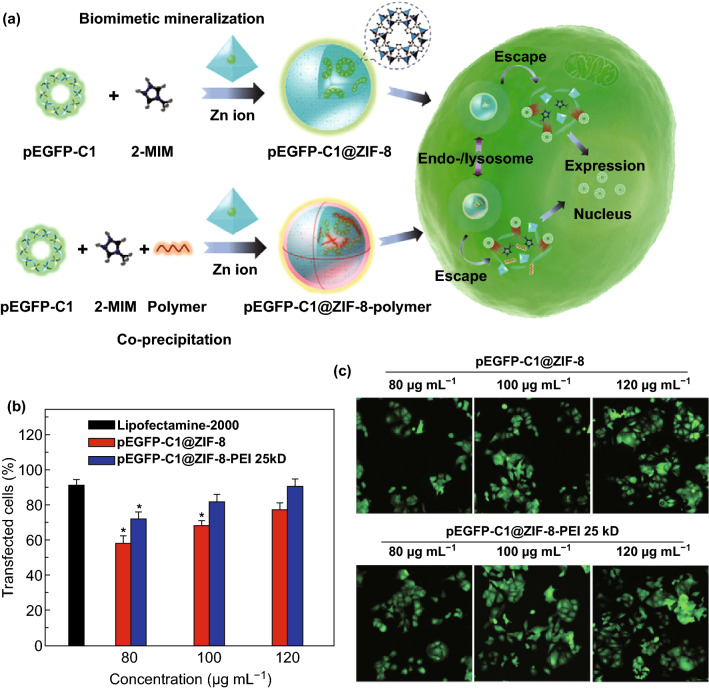
**a** Schematic illustration of the synthesis of pEGFP-C1@ZIF-8 via biomimetic mineralization and pEGFP-C1@ZIF-8-polymer via co-precipitation followed by cellular delivery and expression. **b** Transfection efficacy of pEGFP-C1@ZIF-8, pEGFP-C1@ZIF-8-PEI 25 kD, and lipofectamine-2000 at different concentrations. **c** Representative CLSM images of pEGFP-C1 expression in MCF-7 cells. Reproduced with permission from Ref. [127]. Copyright 2019, WILEY-VCH Verlag GmbH & Co. KGaA, Weinheim

In 2014, Mirkin and coworkers reported the first nucleic acid–MOF nanoparticle conjugates [68]. First, azide-functionalized UiO-66-N_3_ (Zr_6_O_4_OH_4_(C_8_H_3_O_4_-N_3_)_6_) nanoparticles were obtained by solvothermal synthesis. Then, the surface of this nano-MOF was modified with dibenzylcyclooctyne (DBCO)-functionalized DNA through a Cu-free strain promoted click reaction. Compared with nonfunctionalized MOF nanoparticles of comparable size (14 and 19 nm), the synthesized conjugates exhibited higher colloidal stability and enhanced cellular uptake efficiency in the absence of transfection agents. Studies also showed the ability of these nanoparticle conjugates to hybridize with complementary nucleic acids in a sequence-specific fashion, which provided promise for application in intracellular gene regulation. Recently, Tang et al. reported application of ZIF-8 for delivery of plasmid DNA (pDNA) (Fig. 10a) [127]. Capping ZIF-8 by polyethyleneimine (PEI) enhanced loading capacity and binding affinity to pDNA. Efficient gene delivery and expression were observed in MCF-7 cells. In cytotoxicity studies, precultured MCF-7 cells were incubated with different concentrations (80, 100, and 120 μg mL^−1^) of pEGFP-C1@ZIF-8, pEGFP-C1@ZIF-8-PEI 25 kD, and lipofectamine-2000, respectively. After transfection for 48 h, a dose-dependent behavior for both pEGFP-C1@ZIF-8 and pEGFP-C1@ZIF-8-PEI 25 kD was demonstrated by the transfection efficacy (Fig. 10b). The pEGFP-C1@ZIF-8-PEI 25 kD nanoparticles showed a higher transfection efficacy (above 10%) in every dosage. These results were also confirmed by the confocal images (Fig. 10c).

**Fig. 11:**
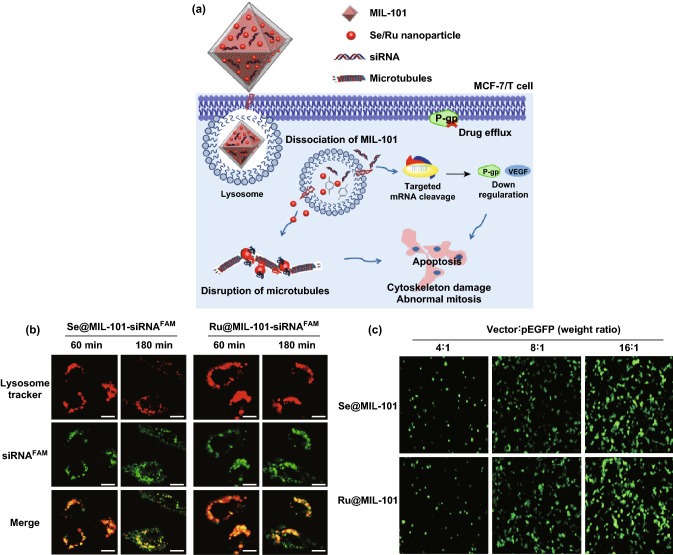
**a** Mechanism of Se/Ru nanoparticles and siRNA co-delivery by MIL-101 for the reversal of drug resistance and induced apoptosis by the disruption of microtubule in MCF-7/T (Taxol-resistant) cancer cells. **b** Time-dependent confocal microscopy of siRNA escaped from endosomes in MCF-7/T cells. Scale bar: 5 μm. **c** Fluorescence microscope images of MCF-7/T cells transfected by Se@MIL-101 and Ru@MIL-101 for 24 h. Reproduced with permission from Ref. [130]. Copyright 2017, American Chemical Society

Small interfering RNA (siRNA) was discovered in 1998, offering a new way to combat resistant cancers [128]. MOFs have been proved as effective nanocarriers for siRNA delivery to protect it against clearance or degradation before taking effect in the target cells. The Lin group reported the first use of MOF nanocarriers for the co-delivery of cisplatin and pooled siRNAs to enhance chemotherapeutic efficacy in drug-resistant ovarian cancer cells (SKOV-3 cells) [129]. siRNA was loaded on the surface of UiO-type Zr-MOF nanoparticles through coordination to Zr_6_ clusters with high loading efficiency (81.6%), while cisplatin prodrug was efficiently encapsulated into the MOF nanoparticles (12.3 wt%). Studies demonstrated the advantages of utilizing MOF nanocarriers to protect siRNAs from nuclease degradation, increase siRNA cellular uptake, and promote siRNA escape from endosomes to silence multidrug resistance genes. Therefore, an order-of-magnitude enhancement of chemotherapeutic efficacy of cisplatin was achieved. Similarly, the Liu group reported the synthesis of MIL-101(Fe) as the nanocarrier to co-deliver pooled siRNAs and selenium(Se)/ruthenium(Ru) nanoparticles to reverse multidrug resistance in Taxol-resistant breast cancer cells (Fig. 11a) [130]. The endosomal escape of siRNA was investigated by confocal laser scanning microscopy. After incubation for 3 h, most of the green fluorescence (siRNA^FAM^) and red fluorescence (lysosome tracker) in the cytoplasm were separated, suggesting the escape of siRNA from the entrapment of endo-/lysosome to accumulate in the cytoplasm (Fig. 11b). The gene transfection efficiency of Se@MIL-101 and Ru@MIL-101 was measured by EGFP transfection assay in MCF-7/T cells (Fig. 11c). The therapy efficacy was enhanced by the silencing of MDR genes and interference of microtubule (MT) dynamics in MCF-7/T cells. Moreover, high targeting specificity to tumor cells, increased antitumor efficacy, and reduced systemic toxicity in vivo were observed. These studies demonstrated the potential of MOF nanoparticles as a novel nanocarrier platform for co-delivery of chemotherapeutic agents and siRNAs to drug-resistant cancer cells.

**Fig. 12:**
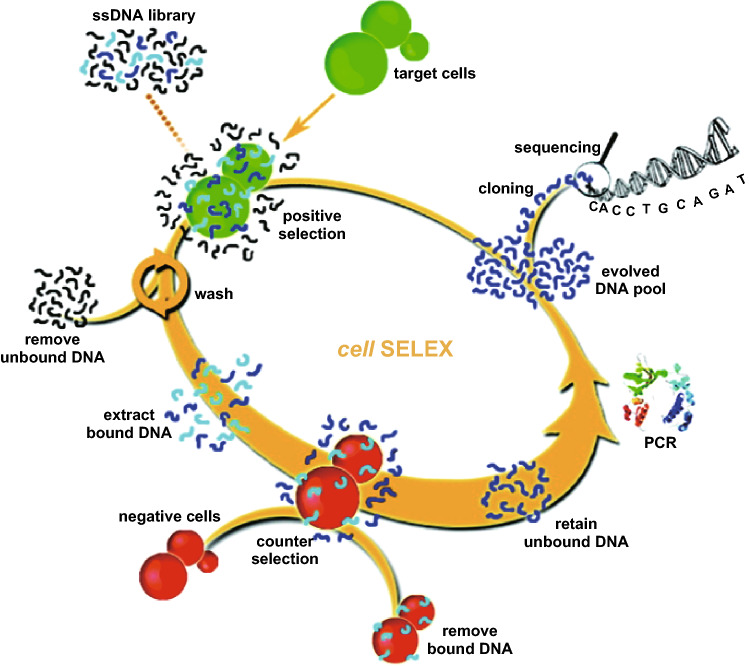
Schematic representation of the cell-SELEX approach for aptamer selection. Reproduced with permission from Ref. [139]. Copyright 2009, American Chemical Society

Nucleic acid aptamers usually consist of short strands of oligonucleotides. These oligonucleotide molecules can be engineered to recognize and bind to specific molecular targets such as small molecules, proteins, and nucleic acids [131–133]. So far, various aptamers have been selected and widely used as effective molecular probes for cancer study based on their high binding specificity and sensitivity, ease of synthesis, improved storage, as well as lack of immunogenicity [134–137]. Particularly, the Tan group pioneered the whole-cell systematic evolution of ligands by exponential enrichment (cell-SELEX) approach for high-affinity aptamer selection [138–142]. This method allows for the selection of aptamers against specific cell lines to accelerate the discovery of biomarkers (Fig. 12). So far, the group has successfully selected a series of aptamers through the cell-SELEX method. For example, aptamers have been selected against leukemia [143], lung cancer [144], and cells infected with the Vacinia virus [145], as well as aptamers specific for phosphorylation epitopes of tau protein [146].

With the development of aptamer selection for molecular medicine, MOF nanocarriers for aptamer delivery have been investigated during the last few years, taking advantage of the unique properties of aptamers. For instance, Fang and coworkers demonstrated that AS1411 aptamer-functionalized UiO-66@AgNCs@Apt can be internalized effectively by target cancer cells (MCF-7 cells) with high selectivity through AS1411-mediated endocytosis [147]. Upon one-pot incorporation of the anticancer drug DOX, this drug delivery system exhibited high capability for targeted delivery and intracellular controlled release, resulting in enhanced antitumor effect in vitro.

**Fig. 13:**
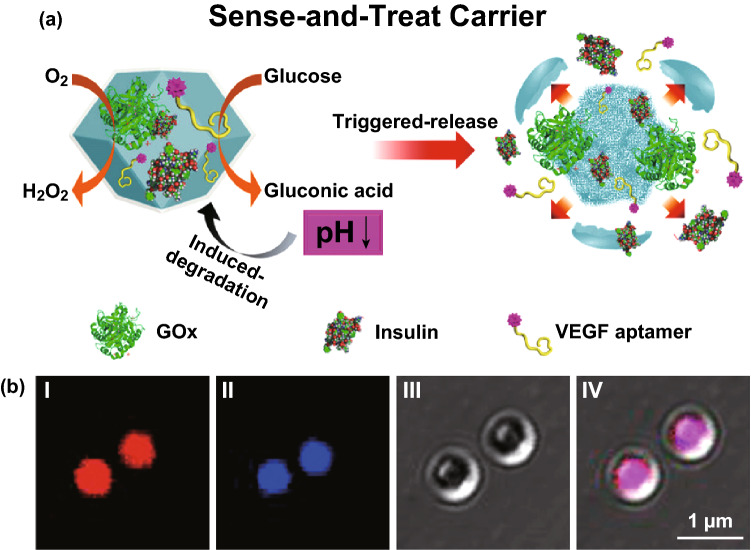
**a** Mechanism of glucose-driven release of VEGF aptamer from ZIF-8 caused by degradation of MOFs under local acidified conditions created by GOx-catalyzed aerobic oxidation of glucose to gluconic acid. **b** Confocal microscopy images of ZIF-8 loaded with Cy3-modified VEGF aptamer (I) and coumarin-functionalized GOx@ZIF-8 (II), and the bright field and merged image of the loaded MOF (III and IV, respectively). Reproduced with permission from Ref. [149]. Copyright 2018, American Chemical Society

Several efforts have been made toward controlled release of drugs utilizing aptamer-functionalized MOF nanoparticles. This was achieved by designing MOFs responsive to different triggers, e.g., ATP and glucose. The Willner group modified the external surface of MOF nanoparticles (UiO-68) with ATP-AS1411 hybrid aptamer in caged configurations [148]. ATP is upregulated in cancer cells, while AS1411 aptamer identifies the nucleolin receptor sites on the cancer cell membrane. In the presence of ATP, the MOFs were unlocked by ATP–aptamer complex formation, releasing the loaded drug molecules (DOX). Experiments revealed high cytotoxic efficacy and highly selective permeation of these dual aptamer-modified MOF nanocarriers into MDA-MB-231 breast cancer cells as compared to MCF-10A normal epithelial breast cells. The group subsequently designed glucose-responsive MOF nanocarriers for controlled release of drugs [149]. ZIF-8 nanoparticles were loaded with glucose oxidase (GOx) and antivascular endothelial growth factor aptamer (VEGF aptamer). Upon GOx-mediated aerobic oxidation of glucose, the products gluconic acid and H_2_O_2_ acidified the microenvironment and caused pH-induced degradation of MOFs to release drugs (Fig. 13a). The VEGF aptamer could potentially inhibit angiogenic regeneration of blood vessels. The loadings of VEGF aptamer and GOx were confirmed by confocal microscopy imaging (Fig. 13b). Panels I and II suggested that the Cy3-modified VEGF aptamer (red) and the coumarin-functionalized GOx (blue) were successfully incorporated into ZIF-8. The Fan group reported immunostimulatory DNA–MOFs (isMOFs) containing cytosine–phosphate–guanosine (CpG) oligonucleotides, which exhibited high cellular uptake, organelle specificity, and spatiotemporal control of Toll-like receptors (TLR)-triggered immune responses [150].

### Proteins

Proteins are macromolecules consisting of one or more long chains of amino acid residues. They serve a large number of functions, such as DNA replication, metabolic reaction catalysis, and molecular transport. Since proteins have large size, charged surface, and environmental sensitivity, it is difficult for proteins to naturally cross cell membranes without losing structural integrity. In order to utilize proteins for therapeutic purposes, MOF nanoparticles for intracellular delivery of proteins have attracted increasing attention in recent years.

For example, Farha et al. selected NU-1000 and PCN-222/MOF-545 nanoparticles as the host for insulin encapsulation [151]. The surface of MOFs was modified with phosphate-terminated nucleic acids for increased colloidal stability and cellular uptake. Compared to the native protein, a tenfold enhancement of cellular uptake was achieved. The Zheng group synthesized a pH-sensitive nanocomposite with a core–shell structure as the drug delivery system [152]. Biocompatible bovine serum albumin (BSA) and DOX (BSA/DOX) core was protected by the ZIF-8 shell. The BSA/DOX@ZIF-8 showed greater antitumor efficacy than that of free DOX against breast cancer cell line MCF-7.

**Fig. 14:**
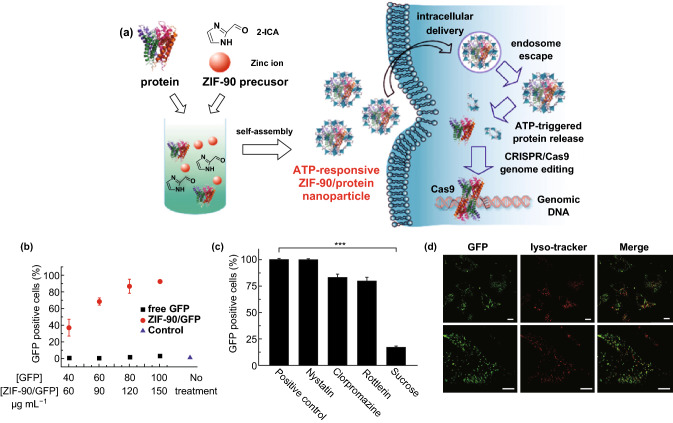
**a** Schematic illustration of the synthesis of ZIF-90/protein nanoparticles and ATP-triggered protein release in the cell. **b** Cellular uptake efficiency of ZIF-90/GFP. **c** Cellular uptake efficiency of ZIF-90/GFP in the presence of different endocytosis inhibitors. **d** CLSM images of HeLa cells treated with ZIF-90/GFP. LysoTracker Red was used for endosome/lysosome staining. Scale bar: 10 μm. Reproduced with permission from Ref. [153]. Copyright 2019, American Chemical Society

Recently, Mao and coworkers have developed zeolitic imidazole framework-90 (ZIF-90) as a general platform to deliver different proteins into the cytosol, independent of their size and molecular weight [153]. Protein encapsulation was performed by self-assembly of imidazole-2-carboxaldehyde, Zn^2+^, and the protein (Fig. 14a). Degradation of nanoparticles to release protein was observed in the presence of ATP. HeLa cells were treated with ZIF-90/GFP nanoparticles for cellular uptake study. According to the flow cytometry analysis, the cellular uptake of ZIF-90/GFP increased proportionally with the concentration of GFP increasing from 40 to 100 μg mL^−1^ (Fig. 14b). Next, different endocytosis inhibitors were selected for pretreatment. Among them, only sucrose reduced the cellular uptake efficiency significantly (down to 17%) (Fig. 14c), indicating that ZIF-90/GFP is mainly internalized via clathrin-mediated endocytosis. After incubation of HeLa cells with 50 μg mL^−1^ ZIF90/GFP nanoparticles, a significant accumulation of GFP in the cytosol was observed by CLSM imaging (Fig. 14d). Furthermore, ZIF-90/protein nanoparticles were used to successfully deliver cytotoxic RNase A for tumor cell growth inhibition, as well as genome-editing protein Cas9 to knock out the green fluorescent protein (GFP) expression of HeLa cells.

Enzymes are a class or proteins that can catalyze many complex reactions in organisms with high selectivity. So far, enzyme–MOF composites have been widely studied for catalysis, sensing, and detection [154–157]. Recently, cellular delivery of enzymes by MOF nanocarriers for cancer therapy has been reported by the Zhou group. These MOFs showed better selectivity and less systemic toxicity than conventional chemotherapy [158]. Tyrosinase was encapsulated into PCN-333(Al) (TYR@PCN-333) to form an enzyme–MOF nanoreactor to activate the cancer prodrug paracetamol (APAP). The reaction generated reactive oxygen species (ROS) and depleted glutathione (GSH), inducing cytotoxicity in drug-resistant cancer cells. Compared to free enzyme, the MOF nanocarrier provided protection against enzyme deactivation and thus extended the antitumor efficacy of TYR@PCN-333.

### Challenges

Although remarkable achievements have been made to apply MOFs for drug delivery, several challenges still exist in this field. First, only limited studies on the kinetics of drug loading and release have been reported so far. Recently, the Horcajada group has demonstrated that the drug loading process is governed by the accessibility of cages in MOFs, while the loading capacity is influenced by the hydrophobicity/hydrophilicity of MOFs and the drug molecules [159]. For instance, the loading rates of hydrophilic acetylsalicylic acid (AAS) and hydrophobic isobutylphenylpropanoic acid (IBU) into UiO-66 are 0.0301 and 0.0295 M h^–1^, respectively. However, higher total drug loading capacity of IBU (35.5%) was observed compared to that of AAS (25.5%). It is worth noting that the solvent may also affect the drug loading rate. According to their studies, both the structure of MOFs and the hydrophobic/hydrophilic nature of the drug molecules could affect the rate of drug release. For example, a faster release of AAS from the open-structured MIL-100 (1 day) was observed, while a slower AAS release from the narrow 1D pore system of MIL-127 (6 days) was detected. Furthermore, a mismatch in hydrophobicity and hydrophilicity could result in a fast drug release. For example, hydrophilic AAS underwent a quick release from hydrophobic UiO-66 (1 day).

Another major challenge for clinical applications of MOF-based DDS is the potential toxicity. However, the existing literature is very limited and insufficient to draw a conclusion about the toxicity of MOF nanoparticles. So far, many in vitro toxicity studies have been conducted on different cell lines, making it very difficult to compare the obtained results. For instance, nanoZIF-8 (200 nm) was evaluated against three human cell lines, namely NCI-H292, HT-29, and HL-60. Results suggested that nanoZIF-8 is nontoxic to these cells [101]. However, in another report, nanoZIF-8 (90 nm) showed cytotoxicity toward HeLa and J774 cell lines [160]. Recently, the in vivo toxicity of nanoscale MOFs has been assessed against zebrafish embryos [161]. The study revealed that the toxicity of MOFs was mainly attributed to the leached metal ions. In contrary, three different Fe(III)-based MOF nanoparticles (MIL-88A, MIL-100, and MIL-88B_4CH_3_) were injected in rats at high doses. The results suggested that these MOF nanoparticles exhibited low acute toxicity and were rapidly sequestered by liver and spleen. According to the studies by Baati et al., the MOF nanoparticles could undergo further biodegradation and elimination in urine or feces without metabolization and causing significant toxicity [162]. In order to reach the clinical development stage of MOF nanoparticles, the performance of MOF-based DDS should be optimized for preclinical evaluation by conducting systematic in vivo studies on their stability, degradation mechanics, and side effects on normal organs.

## Conclusions and Perspectives

During the past few decades, MOFs have been extensively studied for a variety of applications by their well-defined structure, high surface area, high porosity, tunable pore size, and easy functionalization. In particular, exploring MOFs as a nanocarrier for drug delivery in biomedical applications has attracted great interest in recent years. Currently, various molecules have been investigated as the therapeutic agents for disease treatment, such as anticancer drugs, nucleic acids, and proteins. In this review, we summarized four strategies commonly used to functionalize MOFs with therapeutic agents for drug delivery. They include surface adsorption, pore encapsulation, covalent binding, and functional molecules as the building block. The van der Waals interaction, *π*–*π* interaction, and hydrogen bonding are the main forces involved in surface adsorption and pore encapsulation approaches. Functional molecules are covalently bound to the framework through inorganic metal clusters or organic linkers by the covalent binding method. Moreover, functional molecules can be incorporated into the framework as organic ligands. Then, we thoroughly discussed recent progress of biological applications of MOF nanocarriers for drug delivery. Benefiting from unique advantages of MOFs, many drug molecules have been efficiently delivered by MOF nanoparticles. Among them, drugs, nucleic acids, and proteins were selected for discussion in this section.

Despite remarkable achievements made in this field, several challenges remain to be solved. First, although many functionalization methods have been reported, they all possess some limitations. For instance, molecules incorporated by surface adsorption and pore encapsulation tend to leak gradually owing to weak interaction forces. Covalent binding provides stronger interactions, but it requires complex synthetic procedures and may influence the activity of functional molecules. On the other hand, the organic ligands suitable for MOF synthesis are usually rigid and highly symmetrical, which makes it difficult to directly utilize biomolecules as the building block. Such limitations call for the development of advanced functionalization strategies to incorporate a wide variety of potential therapeutic agents into MOFs to explore their clinical applications. Second, the kinetics of drug loading and release, in vivo toxicity, degradation mechanism, and pharmacokinetics of MOF nanoparticles are still under study. Further investigations are required to rationally design MOF–drug conjugates with enhanced biostability, biocompatibility, and therapeutic efficacy. In conclusion, MOFs possess unique properties and show great promise for intracellular drug delivery to treat diseases. In the future, efforts should be focused on overcoming the noted challenges to fully realize the potential of MOFs as drug delivery systems in clinical applications.
